# A Qualitative Study of the Initial Diagnosis and Treatment Experience of Black Patients with Newly Diagnosed Acute Myeloid Leukemia

**DOI:** 10.1177/26892820251403847

**Published:** 2026-02-10

**Authors:** Allison O. Taylor, Laura J. Fish, Kris W. Herring, Jordan Infield, Kimberly S. Johnson, Thomas W. LeBlanc

**Affiliations:** 1 Division of Hematologic Malignancies and Cellular Therapy, Department of Medicine, Duke University School of Medicine, Durham, North Carolina, USA.; 2 Duke Cancer Institute, Behavioral Health, and Survey Research Core (BHSRC), Durham, North Carolina, USA.; 3 Department of Family Medicine and Community Health, Duke School of Medicine, Durham, North Carolina, USA.; 4 Duke Cancer Patient Experience Research Program, Duke Cancer Institute, Durham, North Carolina, USA.; 5 Department of Medicine, Division of Geriatrics, Duke University School of Medicine, Durham, North Carolina, USA.; 6 Geriatrics Research, Education and Clinical Center (GRECC), Veterans Affairs Health Care System, Durham, North Carolina, USA.

**Keywords:** acute myeloid leukemia, bias, health disparities, health equity, qualitative interviews, spirituality

## Abstract

**Purpose:**

Black patients with acute myeloid leukemia (AML) experience disparate outcomes, and factors such as structural racism and differential treatment access have been implicated. We sought to understand the experience of Black patients with newly diagnosed AML to gain insight into their unique experience.

**Methods:**

We interviewed Black patients ≥18 years of age with newly diagnosed AML (diagnosed from 7/1/2021 to 7/31/2022) over Zoom. Interviews were conducted by the same interviewer, with concordance in race between the interviewer and interviewees. A rapid qualitative content analysis was employed.

**Results:**

Ten patients were interviewed, with a median age of 63.5 years (range, 36–82 years). Six were male, married, and treated with a hypomethylating agent. Seven patients had secondary AML. Themes were broadly categorized under (1) the diagnosis and treatment experience, (2) challenges that arose, and (3) coping strategies. Unique findings included the experience of bias during the initial diagnosis period (but not during treatment decision-making discussions), and the overwhelming role of faith/religion/spirituality in coping. Other notable themes included trust, shock and urgent decision making, diverse support systems, physical limitations, the psychological impact, and financial constraints.

**Conclusions:**

Interviewed Black patients with newly diagnosed AML reported bias in the initial diagnosis period, possibly racially motivated, but did not feel this was at play in treatment discussions. The importance of faith/religion/spirituality in coping was also highlighted. Interventions focused on implicit bias training and supporting spiritual needs are critical to improve the experience of Black patients with AML and provide equitable care to all patients with AML.

## Introduction

Acute myeloid leukemia (AML) is the most common acute leukemia and is associated with a poor prognosis, with an estimated five-year survival of only 32%.^1^ Despite recent advances in the treatment landscape, access to care remains unequal, and patients from under-represented racial and ethnic backgrounds continue to have worse outcomes.^2–4^ For example, Black patients are less likely to enroll in clinical trials and undergo allogeneic hematopoietic stem cell transplant, which provides a chance for cure, and therefore experience worse overall survival in comparison with White patients.^5–9^ The underlying drivers of these disparities are poorly understood and remain underexplored.

Preliminary evidence points to multiple barriers, including distal factors (structural racism), intermediate factors (health care access, clinical and molecular characteristics), and proximal factors (treatment patterns and complications).^10^ A better understanding of factors that may influence outcomes along the trajectory of care is needed to develop interventions that address disparities. We were particularly interested in exploring these factors in newly diagnosed Black patients with AML.

A critical aspect of understanding patterns of care to address disparate outcomes is understanding patients’ experiences. Published patient experience studies in AML have included cohorts with a greater representation of White patients.^11,12^ To date, little research has focused on Black patients despite their well-described poor outcomes. Therefore, in this qualitative study, we sought to understand the experience of Black patients with newly diagnosed AML through one-on-one interviews.

## Methods

### Study design, eligibility, recruitment, and consent process

**FIG. 1.**
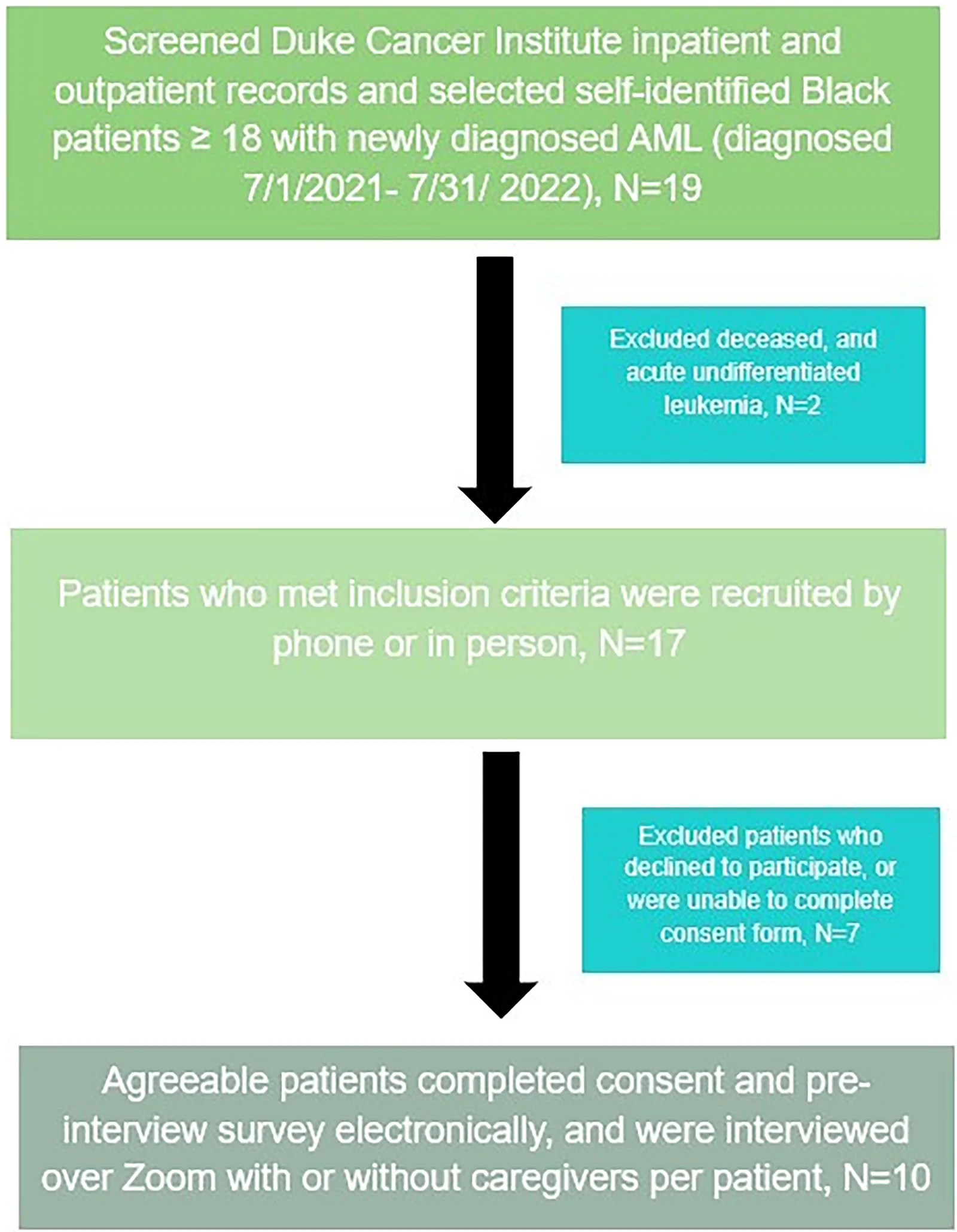
Flowchart of recruitment approach. We screened inpatient and outpatient Duke Cancer Institute records for self-identified Black patients ≥18 diagnosed with acute myeloid leukemia from 7/1/2021-7/31/2022. We identified 19 patients but ultimately excluded patients who were deceased, had a diagnosis of acute undifferentiated leukemia, declined to participate, or unable to consent. Our final cohort included 10 patients.

In this qualitative study, approved by Duke Health Institutional Review Board (Pro00109085), we reviewed inpatient and outpatient records from the Duke Cancer Institute. Using purposive sampling, we selected patients with newly diagnosed AML (i.e., diagnosed from 7/1/2021 to 7/31/2022), who self-identified as Black, were ≥18 years of age, had primary or secondary AML, and were receiving the first line or salvage therapy or had received an allogeneic stem cell transplant. Based on the concept of “information power” to guide adequate sample size for qualitative studies,^13,14^ data saturation was achieved over a one-year period with 19 patients. We excluded two patients (one deceased and one with acute undifferentiated leukemia), and 17 remained. Of this number, five declined to participate (due to poor timing), and two were unable to complete the consent; thus, 10 patients were approached in person or via phone, agreed to participate, completed electronic consent and the preinterview survey, and were interviewed (Fig. 1). The investigative team members were not part of the individual care teams.

### Data collection: Preinterview survey, interview, and electronic medical record clinical data abstraction

The preinterview survey included demographic and socioeconomic factors and the Control Preferences Scale (a validated instrument assessing patients’ preferred role in the treatment decision-making process).^15^ One patient had missing education and control preference data. A.O.T., a Black female physician conducted all the interviews between 4/2022 and 10/2022, thus interviews were race-concordant. Nine were over Zoom and one was in person (hospital). All one-time interviews (three including caregivers per patient preference) lasted approximately 30–45 minutes and were audio recorded and transcribed. Transcripts were not corrected by patients. Questions followed a semistructured guide developed by the study team, focused on the diagnosis and treatment experience, coping, and challenges (see Supplementary Data). Patients were compensated with $25 vouchers at interview completion. Clinical data were collected from the electronic medical record to provide insight into factors relevant to the patient experience: AML status (primary or secondary), treatment regimen, and primary insurance.

### Data analysis

Transcripts were double coded by two authors (J.I. and L.J.F., an experienced qualitative researcher) using a rapid analytic approach to qualitative thematic analysis.^16–18^ This process involved (1) formulation of neutral domain names from each interview question; (2) development of a summary template using domain names; (3) assessment of the usability of the summary template; (4) summarization of transcripts using the summary template; (5) creation of a matrix with domain names and respondent answers; and (6) identification of recurrent themes from the matrix. Patients did not provide feedback on themes.

## Results

### Baseline characteristics

Of the 10 patients interviewed, the median age was 63.5 years (range 36–82). Six were male, and seven had secondary AML. Hypomethylating agents were the most common treatment (six patients) and were combined with Venetoclax in four. Six were married, four had Medicare, and six had more than a high school degree. Seven preferred to share decisions about treatment with their doctor (Table 1).

**Table 1.** Baseline Demographic and Disease Characteristics, and Treatment Decision-Making Role Preferences, *N* = 10

Characteristic	*N* = 10
Age in years, median (range)	63.5 (36–82)
Sex, *N* (%)	
Male	6 (60)
Female	4 (40)
Primary vs. secondary AML, *N* (%)	
Primary	3 (30)
Secondary	7 (70)
Current/recent treatment, *N* (%)	
Hypomethylating agents	6 (60)
Allogeneic hematopoietic stem cell transplant	3 (30)
Clinical trial	1 (10)
Marital status, *N* (%)	
Single	2 (20)
Married	6 (60)
Divorced/separated	1 (10)
Did not answer	1 (10)
Primary insurance, *N* (%)	
Medicaid	2 (20)
Medicare	4 (40)
Private	3 (30)
Veterans Affairs	1 (10)
Education, *N* (%)	
High school degree	3 (30)
Associate degree	1 (10)
Some college/college	5 (50)
Did not answer	1 (10)
Treatment decision making, *N* (%)	
Prefer to make the decision after considering my doctor’s opinion	1 (10)
Prefer to share decision with my doctor	7 (70)
Prefer to leave all decision making to my doctor	1 (10)
Did not answer	1 (10)

AML, acute myeloid leukemia.

### Categorization of themes/subthemes from interviews

The main interview themes fell under three broad categories: (1) the diagnosis and treatment experience, (2) challenges that arose, and (3) coping strategies, **bolded in the paragraphs below** with representative quotes. Additional quotes are in Table 2.

**Table 2.** Themes, Subthemes, and Representative Quotes Identified Related to the Diagnosis and Treatment Experience, Challenges That Arose, and Coping Strategies

Theme	Subtheme	Representative quotes
**The diagnosis and treatment**	**experience**
Experience of bias	-Being dismissed -Negative assumptions related to race	“You’re ok, you’re young you are just having a heavy period”—37-year-old female “Up until that point, no one was really telling me anything… I ask questions, but no one wanted to come out and say what they suspected”—56-year-old male “… because I didn’t react right away [ they thought] I was disengaged, but you got to process things”—68-year-old female
Shock and urgent treatment decision making	-Gradual onset nonspecific symptoms -Rapidity of decisions -Fear and worry	“[ I felt] fatigued, bloated, something was a bit off…”—37-year-old female “[When I heard I had leukemia I thought about] death, because everyone I know who gets cancer, they don’t last too long…, I thought my time was going to be short”—59-year-old male “[I understood that] either you do [ this treatment], or you don’t, if you want to live, you got to do it”—59-year-old male “I was afraid, I was thinking about my family, and I was thinking about providing for them…, what happens next… just a floodgate opened, and all these thoughts kept flooding in my mind…When you hear that word, the C word cancer, it just does something to your psyche”—56-year-old male
Trust and treatment decision making	-Trust in the primary oncologist -Appropriate explanations -Trust in our institution -Role of race in treatment discussions	“… I didn’t know one treatment from another, so I was ready for the doctor to make the whole decision”—83-year-old female “… his professionalism, his bedside manner, really help me a lot, I like a physician with whom I can speak and have a conversation… he was very personable, and I appreciated that”—56-year-old male “I have confidence and trust in my doctors and my doctor, he’s been awesome, I tell you, I don’t think I could have found a better doctor. So, I trust, I really trust, I believe he is leading me in the right direction.”—73-year-old-male “The [explanations about treatment] were very clear, he always explained [the treatment plan] down to the core of everything… that gave me comfort”—73-year-old male “… [our institution] is my hospital, I felt very comfortable”—73-year-old male “I’ve heard good things about… so I chose them and I’m like okay well if I die, I know that they did everything in their power to try to save me…without any bias opinions”—37-year-old female
Sharing with others	-Openness -Privacy	“… you got to enter Black people into the conversation… and start thinking about what they might need which is not necessarily what other people may need…”—68-year-old female “… no, … I’m a very private person, but my closes friends and family know…, I initially did want to keep it to myself so I could process…”—56-year-old male
**Challenges that arose**
Physical limitations	-Physical appearance -Physical abilities Quality of life -Loss of independence	“[The most difficult thing is] lack of mobility, but that’s getting better… and trusting my breathing again”—68-year-old female “… just looking at my new self, the weight gain, that’s really big for me because I was always fit… and I’ve just gained a lot of weight and that just bothers me”—37-year-old female “Relinquishing your independence, even it’s a short duration… it is still difficult to do”—56-year-old male
Psychological impact	-Fear, sadness, and worry -Acceptance	“Well, they tell me that it’s going to take time. A year. A year or two... Which makes me down and depressed cause that’s a long time to deal with this but at least I’m still alive but just sitting around not doing nothing, just bored.”—64-year-old male “… they told me all the stuff that could go wrong… it weighed on my mind; it’s still weighing on my mind”—59-year-old male “I had an entirely different outlook; my heart didn’t jump out… just a calm feeling…”—83-year-old female
Financial constraints	-Increased costs -Loss of income from an inability to work	“Even though we had insurance and Medicaid, we knew that this was not something we could afford on our own, but then through the Leukemia Society, they help us out tremendously… it would have been a big strain on us…”—73-year-old male “I mean it’s pushing me to where I’m telling myself that, in order to save what little bit of my business I have left I’m going to have to get back out there and do what little that I can.”—65-year-old male
**Coping strategies**
Faith/religion/spirituality	-Belief in a higher power -Prayer -Availability of treatment options as a reflection of a higher powers work -Diagnosis strengthening beliefs	“I think it plays a huge part because you need to believe and trust … and you have to trust that those resources that God sent you are good…”—68-year-old female “I prayed to Him, and I gave it to Him.”—83-year-old female “He said we’re going to get you healed. When someone uses that word [its] very powerful, … and indicative of a level of faith, it comforted me to know that I had a physician who was a man of faith.”—a 56-year-old male “I’m spiritual… I believe in God, [He] has ordained, things for our lives…it might not be something that we pick out, but he sees that we are capable, he sees more in us than what we are able to see in ourselves.”—37-year-old female
Support systems	-Diverse	“[In the hospital] the support system has been wonderful and at home the support system has been wonderful”—68-year-old female “I was faced with the decision. I told my friend do you still fight for this? She was the one that influenced me. She said let’s see what [ the doctor has to] say.”—83-year-old female “All the nurses were nice… around the time I was admitted to the hospital, it was Christmas and my daughter’s birthday, so the nurses threw my daughter a birthday party… and that was just huge for me”—37-year-old female “… while going to [our institution] for treatment, I found out that … so many other people, are going through the same stuff with their lives, doesn’t matter about the nationality, the color of [their skin] we are all bound together, and anytime we see each other… we talk to each other… that was a big support to”—73-year-old male

### The diagnosis and treatment experience

#### Experience of bias

In the initial diagnosis period, patients shared experiences **perceived to be a result of bias**, including **feeling unheard, negative racial perceptions and assumptions**. A 37-year-old female described **being dismissed** when she initially presented to care, “… They wrote it off like nothing is wrong… but something was definitely wrong” and alluded to a resulting delay in diagnostic evaluation. Regarding **negative assumptions related to race**, a caregiver of a 64-year-old male mentioned, “There is an expectation that I’m going to come in and be this crazy woman. I think there is a difference when you’re a person of color when you go through this… I think it’s a different experience. There is the expectation that you’re not educated, that you don’t want to fight for what you feel you need to have… it’s different, things that I have felt.”

#### Shock and urgent treatment decision making

Patients experienced a **gradual onset of nonspecific symptoms**, such as fatigue, shortness of breath, and loss of appetite, which they initially dismissed, and many were **shocked by the eventual diagnosis of AML** and commented on **having to make quick decisions** due to the lethality of AML. They also discussed **fear and worry with ** hearing the initial diagnosis of AML, especially after the gradual onset of symptoms. For example, a 73-year-old male mentioned, “It was a complete surprise… [I] was a little fearful, mostly wondering how this could be…” The **rapidity of decisions** surrounding treatment for a chance of survival was apparent from a 56-year-old male who stated, “… [my doctor] told me that it was very rapidly approaching acute myeloid leukemia [and that] … you want to get started with treatment.” Similarly, a 64-year-old male said, “[I was told] you go through this [allogeneic stem cell transplant] or you have twelve weeks [to live].”

#### Trust and treatment decision making

Many patients developed a **trusting relationship with their oncologist** and articulated that the key to this quickly formed relationship was frankness about specifics of the diagnosis, thorough explanations about the diagnosis and treatment options, and the use of language that maintained hope and the feeling that their best interests were at heart. Others also mentioned **trust in the institution** from their positive impression of our institution from prior experiences. Overwhelmingly, patients did not perceive differential treatment based on race. These findings related to trust did not differ by baseline demographic and disease-related characteristics.

**Trust in the primary oncologist** was notable in discussions. A 73-year-old male who said, “I have confidence and trust in my doctor, and my doctor, he’s awesome, he has been awesome, I don’t think I could have found a better doctor, so I really trust, I believe he is leading me in the right direction.” Another 73-year-old male noted that he felt he had **appropriate explanations** to make the best decision and stated, “The more [my doctor] talked to me about it and explained it to me… I said ok, we need to do this… I’m for [transplant] because we got to do something because I couldn’t just do number one [i.e., chemotherapy] and not do anything.”

**Trust in our institution** related to the positive reputation perceived was illustrated by some respondents. A 37-year-old female stated, “… I trusted that they would save my life without any bias opinions, or any thinking that well she is poor, or she is Black, or she doesn’t have enough insurance, I trusted them to just say she’s a person and they were going to save her life.” Similarly, a 68-year-old female stated “[I thought] crap! Until they said it’s the most common kind, and then I [thought] if it’s the most common kind, they’ve done research on it, it may have been in people of other races, not my race, but they have an idea where to start.” This institutional trust came from either **personal experiences** or the **experiences of others** (i.e., family and friends). An 83-year-old female who had prior personal experience mentioned, “…once I took my chemo and radiation, I didn’t have any more trouble for the last 17 years… I have only had good experiences since then.” A caregiver of a 64-year-old male mentioned, “…. I had a friend who had breast cancer, who came to [our institution] to be treated… and she spoke very highly of it.”

Regarding the **role of race in treatment discussions**, our cohort did not feel that this had an impact on treatments. An 83-year-old female stated, “I think I had the best treatment as anybody else….” Similarly, a 73-year-old-male said, “I don’t think [race played a role], I don’t think that came into the picture at all… because the doctor’s mannerisms let me know that he cares….”

#### Sharing with others

When discussing information about their experience with the AML diagnosis and treatment, patients had varied preferences. Some expressed **openness** with family and friends, spurred by a need for support or a feeling of being helpful to others. Others preferred **privacy** as a means of controlling the narrative and managing the time and emotional toll it can take to share the same information. A 68-year-old female noted, “I don’t care who knows, I am an open book… it might help somebody else, and if you can help somebody, you should.” In contrast, an 83-year-old female stated, “[I kept] it on the down low so I wouldn’t have a whole bunch of phone calls, that you got to repeat the same thing over and over…”

### Challenges that arose

#### Physical limitations

Although treatment provided a path to survival, many shared concerns related to changes in their **physical appearance and abilities.** The latter was secondary to fatigue and negatively impacted **quality of life** and **independence**. For example, a 64-year-old male mentioned, “A lot of stuff I can’t do… I can’t go fishing… can’t mow the yard, it’s hard for me to help around the house.” He also said, “… if it weren’t for my wife, I don’t know what I’d do because sometimes I need help taking showers. I can’t cook… there really isn’t nothing I can do. I sit around. I get so tired. I hate to see her doing all the work.” Similarly, a 56-year-old male stated, “… the [second] most difficult thing was dealing with some of the symptoms when I couldn’t do things like go to the gym.”

#### Psychological impact

Many patients expressed **fear, sadness, and worry** with the new AML diagnosis. A 64-year-old male mentioned, “It’s hard when you have all these medications you’re taking; it affects you. Some days you wake up, and you have good days, and some days you wake up and have bad days.” Some discussed the transition to **acceptance** of the diagnosis over time and the resulting motivation. A 37-year-old female said, “I am always trying to be a step ahead of the game [with treatment], like if this isn’t it, let’s see what else there is.”

#### Financial constraints

Patients discussed **increased costs** and a **loss of income from being unable to work**. A 65-year-old man stated “… with a business, if you don’t have anything coming in, that puts a little pressure on you because bills got to be paid, one way or other….” After being told he would be unable to work for a period of time, a 56-year-old male stated, “…I expected a month or two, and he told me it would be a while… [and] the average patient had to apply for disability because they had to take a leave of absence from work for about a year… it was a lot to process, I could feel the moisture come into my eyes….”

### Coping strategies

#### Faith/religion/spirituality

The prominent role of faith/religion/spirituality was clear and expressed by nine out of ten patients as a source of support. These patients did not differ by baseline demographic and disease-specific characteristics. Patients highlighted **belief in a higher power, prayer, the availability of treatment options as a reflection of the higher power’s work, and the AML diagnosis strengthening beliefs.** A 65-year-old male said, “… believing in something is always good. You always want to be a part of something.” A caregiver of a 64-year-old male mentioned, “… we keep prayer in our life. We do that every day, sometimes 2–3 times, and it has helped a whole lot.” A 69-year-old female mentioned, “… my God and my doctors. Everything goes together. They work together. You use the resources He provides, or you don’t.” Finally, a 73-year-old stated, “I know in whom I believe, and I know God has brought me through many things, and I know that he didn’t bring me this far to leave me….”

#### Support systems

Patients highlighted their **diverse support systems,** including friends, family, neighbors, church members, clinical teams, the Leukemia/Lymphoma Society (Blood Cancer United), and other patients. This support was highlighted by patients across the board with no differences with respect to age, treatment type, primary vs. secondary AML, educational, marital, and insurance status. Referring to his primary oncologist, a 73-year-old male said, “Every morning… he came to the room at 6 o’clock in the morning… I don’t know of many doctors that would do that….” A 56-year-old male mentioned, “There has been an outpouring of love, prayers, and affection from family, friends, and my fraternity brothers.” He also stated, “When I was in the hospital, the nurses were amazing and took excellent care of me… and they would make shakes for me [when I didn’t feel like eating].”

## Discussion

To our knowledge, this study is the first to exclusively examine the lived experience of newly diagnosed Black patients with AML. Our cohort described their experiences related to (1) diagnosis and treatment, highlighted (2) challenges that arose, and described (3) coping strategies. Some unique aspects of the experience shared by patients include the **overwhelming role of faith/religion/spirituality in coping** and the **experience of bias** in the initial diagnosis period. Several themes were consistent with prior AML patient experience work, including **shock and urgent decision making**, the role of **trust in the institution and their oncologist** in treatment discussions and decision making, the importance of **diverse support systems**, **physical limitations, financial constraints**, and the **psychological impact** of the diagnosis; thus, our work adds to the body of literature.

Patients noted bias during the initial diagnosis period. They did not explicitly characterize the basis of this bias, but a caregiver felt it had racial underpinnings. Racial bias in the delivery of oncological care is pervasive and, unfortunately, has been well described. Black breast, prostate, and colorectal cancer survivors have described longer wait times, not getting access to all treatment options, and poor interactions at multiple levels (desk staff, physicians, and insurance administrators).^19,20^ Interestingly, our cohort did not feel like racial bias played a role in the receipt of AML therapies, even on explicit questioning, and felt that once diagnosed, they received equal care. Three possible explanations are as follows:

We hypothesize that, in comparison with solid tumors, with the acute nature of most AML presentations, the need for urgent initiation of treatment, and the more specialized and acute tertiary care-based treatment of AML, may minimize bias related to the receipt of AML therapy, in part due to the standardized approaches to the management of AML. At presentation, all patients receive a bone marrow biopsy with guideline-based ancillary testing to aid in risk stratification and evaluation of targetable mutations. This is followed by the decision between intensive versus less-intensive therapy backbone based on functional status. The abovementioned features of AML can impact the experience of patients in other ways, and in qualitative work by LeBlanc et al., shock/suddenness, uncertainty, poor clinician communication, and challenges with processing information were some of the concerns mentioned by patients.^12^

The initial diagnosis period may be more prone to bias, unlike the treatment phase, due to differences in where there is initial suspicion for AML. Particularly for patients with a primary AML diagnosis who are more likely to be present to an emergency room or urgent care that could have a lower suspicion for AML, compared with those with secondary AML who follow closely with an oncologist. Although bias can be at play, it is also important to consider the challenges that can arise with diagnosing rare illnesses. In a rare disease like AML, patients may not present acutely but rather with vague symptoms such as fatigue and poor appetite, which can often be misattributed.^21,22^ Depending on the site of presentation, there can also be varying awareness/consideration of a rare hematological malignancy like AML.^23^ Additionally, there can often be delays in the diagnostic workup for AML if a patient presents with cytopenias but no blasts, with long wait times for hematology evaluations and bone marrow biopsies, which can make the diagnosis challenging.^24^

Trust could have played a role in patients not reporting experiences of racial bias due to the development of early and trusting relationships between patients and their oncologist in the setting of urgent treatment. Patients may have been reluctant to report mistrust despite race concordance between the interviewer and interviewees due to fear that any concerns shared could affect their ongoing treatment, although the interviewer was not a member of the treatment team. Key drivers of medical mistrust include patient–provider discordance (race, ethnicity, primary language), experienced/perceived/anticipated discrimination, negative view of Western medicine, and experienced/perceived low-quality care.^25^ Trust and effective communication are critical to treatment decision making.^12,26^

Among Black patients, high rates of medical mistrust in the receipt of health care have been well described.^27^ A cross-sectional survey from the Health Information National Trends Survey of 6252 adults in 2022 noted medical mistrust among 62.3%, and 67.8% of these respondents were Black.^28^ A qualitative study focused on factors impacting COVID-19 vaccine decision making in Black patients highlighted that mistrust was rooted in prior experiences with discrimination, feeling dismissed by their health care provider with provider insistence on specific treatments, and feeling like just a number.^29^ In audio-recorded cardiology encounters prior to a communication coaching intervention, Black patients had a statistically significantly lower median number of trust statements and a decreased likelihood of fully open conversations.^30^

In oncology, medical mistrust is also pervasive. In an observational study of men with prostate cancer, African American race, fewer years of education, seeing clinicians for a more extended period (>3 months), and lower perceptions of group interdependence were associated with higher levels of mistrust.^31^ Similarly, in a qualitative study examining the experiences of Black and White multiple myeloma patients and their caregivers (dyads), Black dyads expressed knowledge of the Tuskegee Study, the related legacy of mistrust in the health care system, and thus the need for self-advocacy in receipt of myeloma care.^32^ Overall, medical mistrust is more prevalent among Black patients across oncology and nononcology specialties and is influenced by multiple factors.

To address the possible reluctance to share issues related to mistrust that could have been at play in our work, future works could incorporate: (1) anonymous surveys, (2) longer term follow-up to develop longer term relationships, which could make patients more comfortable sharing, and (3) a multidisciplinary research team that includes race-concordant social workers and nurses who patients may feel more comfortable sharing negative experiences with compared with physicians.

The importance of faith/religion/spirituality in coping for cohorts of patients from different racial and ethnic backgrounds has been discussed in prior qualitative work in both solid tumors and hematological malignancies, including AML.^33,34^ For example, Newcomb et al. evaluated the role of approach-and avoidant-oriented coping techniques in 360 patients with hematological malignancies hospitalized for autologous or allogeneic hematopoietic stem cell transplants. Forty-four percent reported using approach-oriented coping techniques, including but not limited to religion.^35^

In our work, 9 out of 10 patients endorsed faith/religion/spirituality as a critical coping mechanism, and the overwhelming importance of faith/religion/spirituality is consistent with prior studies in Black patients with different malignancies.^36^ In a qualitative study of Black patients with both solid and liquid tumors, notable themes that emerged related to faith/religion/spirituality were God as a helper, God as a healer, the importance of prayer, and Bible/Scripture study.^34^ Similarly, among Black breast cancer survivors, religiousness, spirituality, and God were named as a source of strength throughout the initial diagnosis, treatment, and recovery.^37,38^ Although the prominent role of faith/religion/spirituality is not unique to our cohort, the overwhelming endorsement of its role in coping highlights opportunities to develop targeted interventions to support faith and spiritual needs.

Like prior works, our cohort described the shock of the AML diagnosis, the need for urgent treatment decision making, physical and psychological implications, and the need for diverse support systems. In these studies, AML patients have cited negative emotional responses related to their diagnosis (fear, shock, powerlessness, uncertainty), and the importance of support from family, friends, and health care professionals.^11,12,33,39,40^ Nissim et al. described the initial traumatic experience of the AML diagnosis and hospitalization, as well as the resulting psychological response to the inpatient induction treatment.^41^ Similarly, Richardson et al. mentioned that the top two worries expressed by patients and their caregivers were related to “the possibility of dying from AML” and “long-term side effects of treatments.”^42^

For financial constraints noted in our work, similarly, in a single-center study of patients with acute leukemia (acute myeloid and lymphoid leukemia), roughly half (54%) of the patients surveyed over 6 months reported financial toxicity.^43^ A qualitative study of AML patients in remission in Australia described the burden of AML-attributable costs, having to accommodate for the impact of AML on paid work, the financial strain from AML, and concerns about the future and future familial financial burden.^12,44^

## Limitations

There are some limitations to this research worth mentioning. First, our sample size of ten after exclusions was small, but our sampling technique was based on the concept of informational power, common in qualitative research. Given the rarity of AML in general and in patients from underrepresented racial and ethnic groups, we believe this was a reasonable number of patients. Second, the experiences of patients we interviewed may not be generalizable to the experiences of all Black patients. The goal of qualitative research is not to achieve generalizability but to understand the experience and perspectives of a particular group. Third, patients may not have felt comfortable sharing negative experiences, since they were interviewed by a race concordant clinician at the institution where they received care, even though the interviewer was not part of their treatment team. Despite limitations, this work is novel in describing specifically and deeply for the first time the unique experience of Black patients with newly diagnosed AML.

## Conclusion

Black patients with AML have worse outcomes than their White counterparts. Inequitable access to treatments, structural racism, and racial bias are just some of the factors that lead to these disparate outcomes, but individual patient experience can have an impact on outcomes. Thus, our work focused on the experience of Black patients with newly diagnosed AML. Exploring the initial diagnosis and treatment period, understanding challenges that arose, and coping strategies employed in a population where there has been limited patient experience work highlighted the experience of bias during the initial diagnosis period and the vital role of faith/religion/spirituality in coping. Surprisingly, patients expressed trust in their oncologist, which was instrumental in treatment decision making.

Our findings emphasize the continued need for interventions to support Black patients with serious illnesses like AML to improve health care equity by supporting faith/religion/spirituality, reducing diagnostic bias, and continuing to build trusting relationships. To support faith/religion/spirituality, future studies should use mixed methods to gain perspectives from oncologists, patients, caregivers, and spiritual/religious leaders to jointly develop programs/initiatives that can be integrated into the health system. To reduce bias and continue to build trust, clinicians should continue to engage in implicit bias and communication training at prespecified and regular intervals, for example, quarterly.

## Authors’ Contributions

Conceptualization: A.O.T.; Methodology: A.O.T., L.J.F., K.S.J., and T.W.L.; Investigation (qualitative interview data collection): A.O.T.; Formal analysis: A.O.T., L.J.F., J.I., K.S.J., and T.W.L.; Writing—original draft preparation: A.O.T.; Writing—review and editing: A.O.T., L.J.F., K.S.J., J.I., K.W.H., and T.W.L.; Project administration: K.W.H.; Funding acquisition: A.O.T., L.J.F., and T.W.L.; Supervision: T.W.L.

## Supplemental Material

sj-docx-1-pmr-10.1177_26892820251403847 — Supplemental material for A Qualitative Study of the Initial Diagnosis and Treatment Experience of Black Patients with Newly Diagnosed Acute Myeloid Leukemia Supplemental material, sj-docx-1-pmr-10.1177_26892820251403847 for A Qualitative Study of the Initial Diagnosis and Treatment Experience of Black Patients with Newly Diagnosed Acute Myeloid Leukemia by

## Abbreviations

AML: Acute myeloid leukemia
COVID-19: Coronavirus disease 2019
N: number
vs: versus
