# Supplementary material for: A Qualitative Study of the Initial Diagnosis and Treatment Experience of Black Patients with Newly Diagnosed Acute Myeloid Leukemia
Source: Palliat Med Rep. 2026 Feb 10;7:26892820251403847. doi: 10.1177/26892820251403847 (PMC13539012; doi:10.1177/26892820251403847)
Supplement: Supplementary file 1 [file sj-docx-1-pmr-10.1177_26892820251403847.docx]

**Interview Guide**

Thank you for taking time to speak with me today. I am interested in learning about your experience following a new acute myeloid leukemia (AML) diagnosis. Thank you so much for allowing me to talk with you about how things have been since your diagnosis.

Is it okay for me to audio record our discussion?

Please know that what we discuss will not be shared with anyone outside of our research team and none of the information will be used to identify you. If at any point, you want to take a break or stop the interview, please do not hesitate to let me know. If there is any topic that you do not want to talk about, that’s okay too, just let me know. Do you have any questions before we begin?

**I would like to ask you some questions regarding when you found out about your leukemia diagnosis, as well as your experience at our institution.**

1. When did you start to not feel right? Was there a particular time or event that you remember when something was not right?
2. The next step was going to see a doctor, can you tell me about that? What were you thinking and feeling when you went to the doctor?

- Did any prior experiences you had with doctors or healthcare impact your decision to go to the doctor?

3. When did you find out you had leukemia and how did the diagnosis make you feel?
4. When did you first come to our institution for leukemia treatment?

- Why did you choose our institution to get your treatment?
- Had you heard anything about our institution before you came here?
- Discuss anything positive or negative and give specific examples.

5. Can you talk to me about your experience with our institution so far?

- What has been good or not good about your experience? Explain and give specific examples.

**Next, I would like to ask you more specific questions about your personal experience with AML.**

6. What challenges (if any) have you had to manage because of your new diagnosis?

- Have you felt overwhelmed or scared?
- Do you feel like you have a strong support system?
- Has your spirituality/belief in a higher power helped you through the experience?
- Have you had any financial challenges because of your AML diagnosis?
- Do you feel like you have lost some of your independence or have had to rely more on others? If so, how has this made you feel?
- Did you feel pressure to hide this new diagnosis from people around you?

How have you addressed these challenges? Are there any additional challenges you would like to discuss?

**Now we are going to talk about the different AML treatments that were discussed with you.**

6. Do you feel like everything about available AML treatments was explained to you in detail?
7. Were you offered a transplant?
8. Was it made clear to you that you had a choice with respect to your treatment choice?
9. When you heard about the different treatment options, do you feel the all the risks and benefits were broken down to a level you could understand? Were you able to ask your cancer doctor all the questions you had about the different treatment options? Did your cancer doctor use any visual aids and/or materials to help you understand the treatment options?
10. Did you feel like you could express things important to you in life in conversations about treatment with your cancer doctor?
11. Ultimately, did you feel like the decision about the treatment plan was made with you and your cancer doctor together?
12. Do you think anything could have been done differently regarding the initial presentation of treatment options?

**The following questions are focused on living with AML and differences in outcomes based on specific factors.**

13. What resources available at our Cancer Center were offered to you to provide support?
14. Recent research has shown real differences in outcomes in AML patients based on different factors, what do you think might be causing these differences?

- Has your distance from our institution had an impact on your treatment?
- Do you live in a rural or urban community? If your live in a rural community, has living there impacted your treatment?
- Do you think your race impacted your care?
- Do you think the race of the person who first told you that you had AML impacted your treatment decision?
- Do you feel like your income or education impacted your treatment?
- Do you feel like your employment or insurance status impacted your treatment?
- Did the level of training of the person who first told you about your AML diagnosis have an impact?

**Lastly, is there anything we did not discuss that you would like to talk about?**

**Do you have any questions for me?**

**Thank you for your time. Please do not hesitate to reach out if you have any further thoughts or questions.**
